# Korean version of the MNREAD acuity chart

**DOI:** 10.1038/s41598-024-57717-4

**Published:** 2024-03-28

**Authors:** Ungsoo Samuel Kim, Keun Soo Kim, Yoon-Shin Kim

**Affiliations:** 1 Department of Ophthalmology, Chung-Ang University, Gwangmyeong Hospital, Deokan-Ro 110, Gwangmyeong-si, Gyeonggi-do 14353 South Korea; 2 Korean Low Vision Society, Seoul, South Korea; 3 DMC Good Eye Clinic, Seoul, South Korea; 4 Department of Korean Language Education, College of Education, Incheon National University, Incheon, South Korea

**Keywords:** Visual acuity chart, MNREAD chart, Low vision, Health care, Medical research

## Abstract

To investigate the efficacy of the Korean version of the Minnesota low vision reading chart. A Korean version consisting of 38 items was prepared based on the MNREAD acuity chart developed by the University of Minnesota. A linguist composed the representative sentences, each containing nine words from second and third grade levels of elementary school. Reading ability was measured for 20–35-year-old subjects with normal visual acuity (corrected visual acuity of logMAR 0.0 or better). The maximum reading speed (words per minute [wpm]) for healthy participants, reading acuity (smallest detectable font size), and critical print size (smallest font size without reduction of reading speed) were analyzed. The average age of the subjects was 28.3 ± 2.6 years (male:female ratio, 4:16). The average reading time for 38 sentences was 3.66 ± 0.69 s, with no differences in the average maximum reading speed between sentences (p = 0.836). The maximum reading speed was 174.2 ± 29.3 and 175.4 ± 27.8 in the right and left eye, respectively. Reading acuity was measured as logMAR 0.0 or better in 80% of the cases. All subjects showed a critical print size of 0.2 logMAR or better. The overall reading ability can be measured using the Korean version of the MNREAD acuity chart, thereby making it useful in measuring the reading ability of those with Korean as their native language.

## Introduction

Visual acuity is a widely used essential component of evaluating visual function. More precisely, reading skills are more commonly used in real life than identifying individual letters. However, as conventional visual acuity charts are composed of individual letters, reading skills cannot be evaluated. Moreover, since various dysfunctions, such as visual field defects, loss of contrast sensitivity, or ocular movement abnormalities, can affect reading speed, it is necessary to investigate reading speed in patients with low vision due to corneal opacity, cataracts, retinal disorders, or optic neuropathies^1–3^.

Reading charts, such as the Bailey-Lovie word chart, Colenbrander English continuous text, RADNER reading acuity chart, and MNREAD chart have been introduced by the International Council of Ophthalmology^4,5^. In 1989, Mansfield and Legge introduced Minnesota Low-Vision Reading chart, which is based on the computer to measure reading speed. Afterward, This test was redesigned in print form in 1993, and it was called the MNREAD Acuity Chart. The MNREAD Acuity Chart consists of sentences from second- and third-grade reading materials, which contain 60 characters and range from 1.3 to − 0.5 logMAR. This chart can measure reading ability, including reading acuity, critical print size, and maximum reading speed and reading accessibility index^6^. English, German, French, Spanish, Dutch, and Turkish versions of the MNREAD acuity chart are currently available.

The Korean alphabet, called Hangul, comprises two or three parts (initial consonant, middle vowel, and optional ending consonant letters [e.g., for 강, ㄱ is the initial consonant, ㅏ is the middle vowel and ㅇ is the ending consonant). Therefore, it is difficult to develop a Korean version of the visual acuity chart because these characters were not based on any alphabet, unlike Chinese or Japanese characters^7^. Therefore, we aimed to investigate the efficacy of the Korean version of the reading acuity chart based on the MNREAD acuity chart.

## Methods

**Figure 1:**
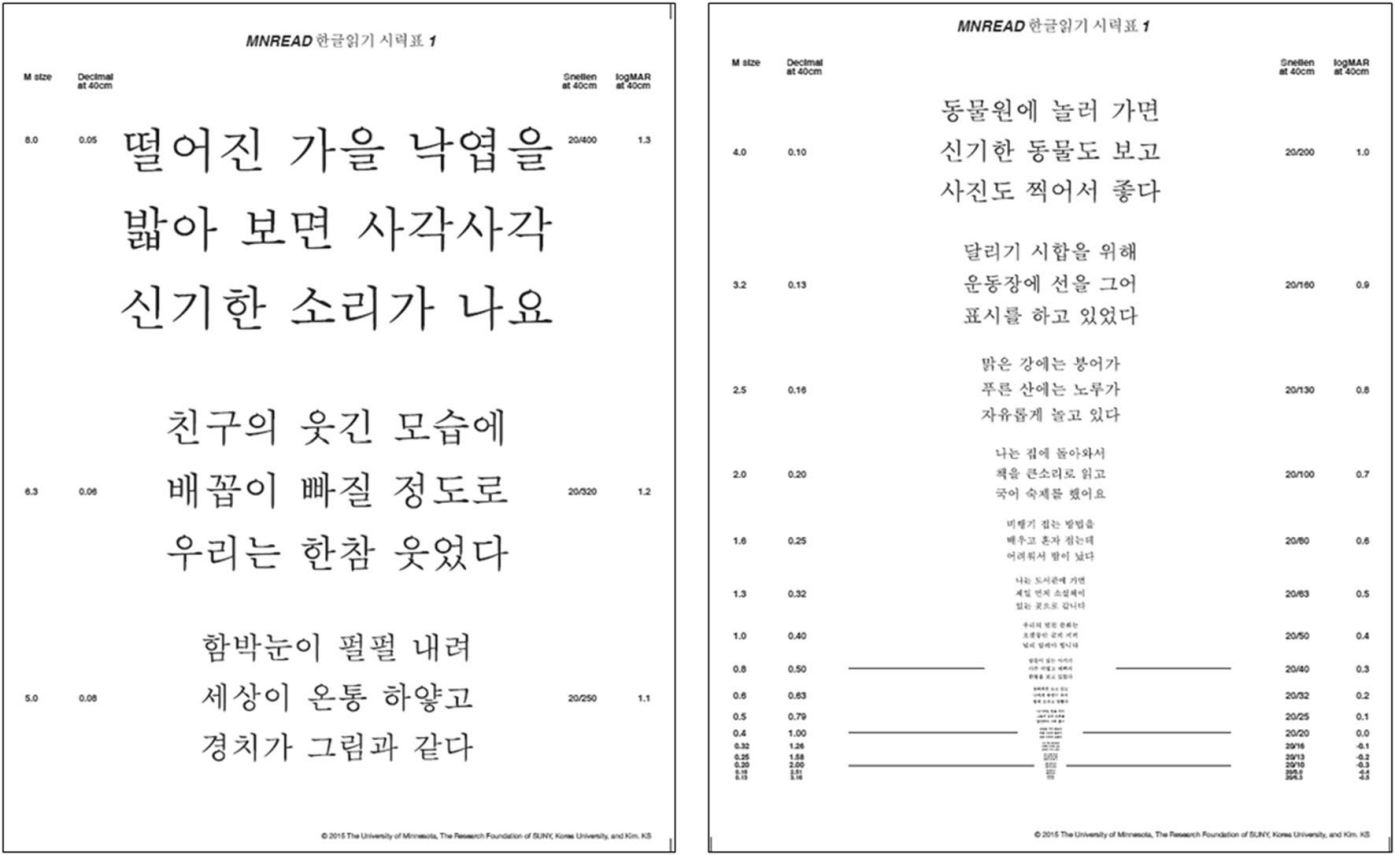
Korean version of the MNREAD acuity chart. For example, the contents of Korean are as follows; 8 M size; When you step on fallen autumn leaves, you hear a strange crunching sound. 6.3 M size; We laughed so much that our belly button fell out at the funny look of our friend. 5 M size; The snow is falling, the whole world is white, and the scenery is like a painting. 4 M size; It's nice to go to the zoo to see strange animals and take pictures.

Representative sentences were obtained from second- and third-grade primary school materials by a linguist (YSK). Thirty-eight items were prepared with similar maximum reading speeds for the right and left eyes. One sentence consisted of nine words, and the font used was MyeongjoChe (Fig. 1). Similar to other language versions of the MNREAD chart, the print size ranged from 1.3 to − 0.5 logMAR. The height of the characters was designed according to the English version of MNREAD chart. This study (no.: P01-202111-21-012) was approved by the institutional review board, and was conducted in accordance with the tenets of the Declaration of Helsinki.

Twenty healthy adults who visited the clinic for refractive surgery from February to July 2021 were included. Complete ophthalmologic examinations, including slit-lamp, fundoscopy, visual field, and refraction tests, were performed. The exclusion criteria were as follows: age > 35 years, far vision < logMAR 0.0, near vision < logMAR 0.0, reading disabilities, visual field defects, and low educational status.

The Korean chart was commissioned by the Precision Vision Company (Woodstock, USA) and created according to the guidelines of the MNREAD acuity chart. The test was conducted under the following conditions: the chart’s white background’s luminance was 80 cd/m^2^, and the testing distance was 40 cm. Each sentence was to be read out aloud as quickly and accurately as possible. The next lines were covered so that the subjects could not preview those sentences. Reading acuity was determined by the smallest print size that the patient could read without making errors, and was calculated as follows: 1.4 − (sentences × 0.1) + (wrong words × 0.011). The maximum reading speed (words per minute [wpm]) was defined as the maximum reading speed for prints larger than the critical print size, and was calculated as follows: speed = 60 × (9 − wrong words)/reading time in seconds. The critical print size was the smallest print that the patient could read with maximum speed. Two panels on the left and the right were created and compared and analyzed.

Data were analyzed using SPSS 18.0 for Windows (SPSS Inc., Chicago, IL, USA). Reading acuity was analyzed using the intraclass correlation coefficient (ICC).

### Ethics approval and consent to participate

The study, No; P01-202111-21-012, was approved by the Korea National Institute for Bioethics Policy (KoNIBP) and conducted in accordance with the tenets of the Declaration of Helsinki. The KoNIBP completely waived the requirement to obtain informed consent, and instead approved a consent procedure, which does not include some or all of the required elements of informed consent, provided that the research involves no more than minimal risk.

## Results

**Figure 2:**
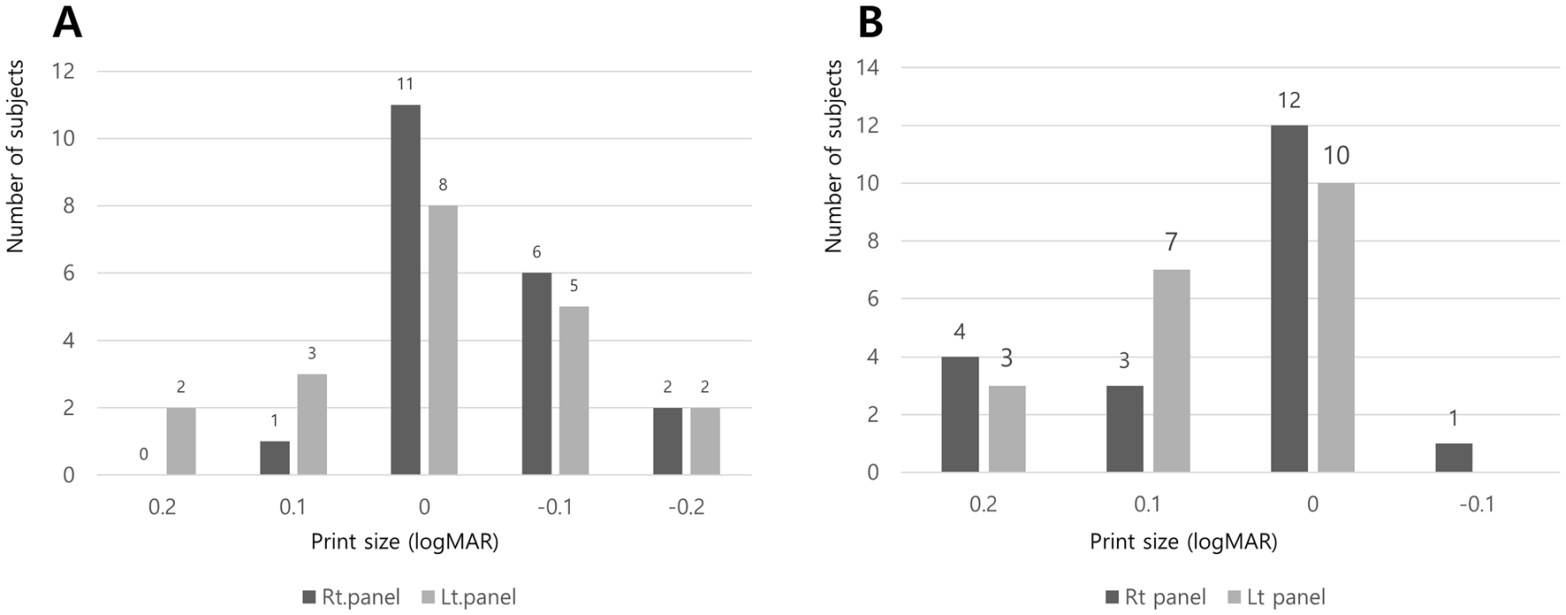
Reading acuity and critical print size in the Korean version of the MNREAD acuity chart (x-axis: logMAR acuity; y-axis, number of participants). (**A**) Reading acuity, (**B**) critical print size.

**Figure 3:**
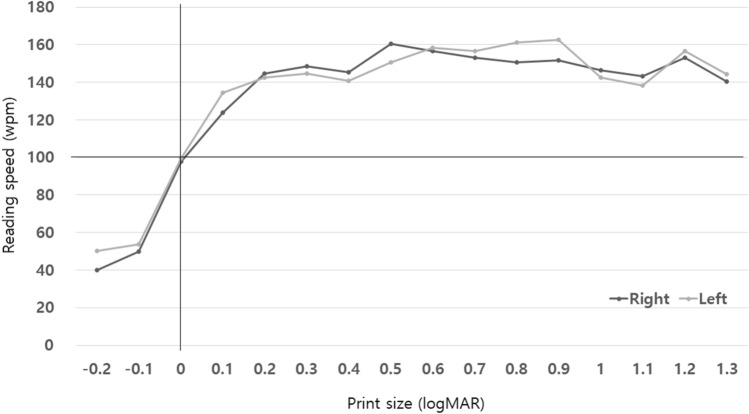
Reading speed (words per minute [wpm]) using the Korean version of the MNREAD acuity chart.

The average age of the experimental group was 28.3 ± 2.6 years (male:female ratio = 4:16). Reading acuity was measured as logMAR 0.0 or better in 80% of cases, the smallest size readable (reading acuity) was found to be the most frequent at 0.2 print size (Fig. 2A). The Maximum reading speed was 174.2 ± 29.3 and 175.4 ± 27.8 in the right and left eye, respectively. Reading speed plateaued at around 0.2 logMAR (Fig. 3). Additionally, there was no significant difference between the right and left panels (*p* = 0.372). However, there were significantly different maximum reading speeds with respect to the MNREAD acuity chart (*p* < 0.001, intraclass correlation coefficient). Regarding the critical print size, approximately half of the group had a 0.0 logMAR letter size (Fig. 2B), while all subjects showed a critical print size of 0.2 logMAR or better. The average reading time for the 38 sentences was 3.66 ± 0.69 s, with no difference in the average reading speeds between sentences (*p* = 0.836) (Table 1).

**Table 1:** Intraclass correlation coefficient.

	Single measures	Average measures	P-value
Right panel	0.335	0.910	< 0.001
Left panel	0.178	0.813	< 0.001

## Discussion

This study provides represented data for the Korean MNREAD chart in the young adult, and the present data, including reading acuity, maximum reading speed, and critical print size, could be representative values to compare normal people and low vision patients in Korean populations.

MNREAD acuity charts have been developed in different languages^8–10^. Each language has its own characteristics. In terms of Korean consonants (e.g., ㄷ vs. ㄸ), each letter has a different level of difficulty of recognition. ETDRS also has the same problem as it contains five letters per line, making it difficult to increase reproducibility. To minimize this problem, the letters in the Korean version of the MNREAD acuity chart were created by a linguist using second- and third-grade materials. Thus, our calculated reading acuity and the maximum reading speed (0.03 ± 0.10 and 174.4 ± 30.0 wpm, respectively) were similar to those of the English version of the MNREAD acuity chart (0.04 ± 0.11 and 153 ± 24 wpm). However, the calculated critical print size was better than that of the English version but identical to the Persian version (English version, 0.35; Persian version; − 0.068 and 0.056). This difference could result from the different shapes and compositions of letters in the Korean version (Table 2).

**Table 2:** Comparison of results among different languages in MNREAD acuity chart.

	Korean (present study)	English^11^	Persian^8^	Turkish^9^	Portuguese^10^
Maximum reading speed (words/min, WPM)	174.2 ± 29.3 (Right panel) 175.4 ± 27.8 (Left panel)	200 ± 25 (16–40 years)	137 ± 5.31	191.50 ± 32.19 (chart 1) 190.55 ± 27.35 (chart 2)	197.8
Critical print size	0.06 ± 0.08	0.21 (16–40 years)	1; − 0.068 ± 0.047 2: − 0.056 ± 0.051		
Reading acuity	0.03 ± 0.10	− 0.18 (16 years)	1; − 0.13 ± 0.011 2; − 0.14 ± 0.018		

The maximum reading speed was around 174 wpm. The average reading speed was around 200–210 in English version and around 190 in Turkish version. The reason of the difference of reading speed is thought to be that Hangul has words with three consonants. Unfortunately, in the present study, the maximum reading speed was not the same for different people. Maximum reading speed varied depending on the reading tendency of each person. According to the instructions, reading as fast as possible was a rule. Nevertheless, because some people read rather slowly and some read very fast, the differences in reading propensity could not be ignored. Differences in maximum reading speed have also been reported in other studies^9,12^.

This study had some limitations. We did not compare the changes in the maximum reading speed, critical print size, or reading acuity in patients with low vision or old patients. Even normal people show differences according to age, so care should be taken in interpretation^11^. Therefore, further research is needed on comparing normal people and patients with low vision using the Korean version of the MNREAD acuity chart. The most important part is the consistency of each sentence^13^, and other Turkish or Greek versions were produced based on conditions similar to this study. In this study, sentences of second and third grade primary school were selected, and although the pattern is consistent to some extent as shown in Fig. 3, further research is needed on the reading ability between these sentences. Finally, we did not investigate the reading accessibility index because the number of subjects was not large enough to determine the parameter corresponding to the denominator of the formula^6^. In the future, we plan to establish a reference point of reading accessibility index through large-scale research.

In conclusion, the Korean version of the MNREAD acuity chart shows similar results to the existing chart. Therefore, this chart is expected to be useful for measuring the reading ability of Korean patients and for treatment evaluation.

## Data Availability

The datasets generated and/or analyzed during the current study are not publicly available as they are not digital data; however, they are available from the corresponding author on reasonable request.
